# Patients with anti-SAE+ dermatomyositis display refractory and difficult-to-treat skin manifestations: case series from two Italian cohorts and review of literature

**DOI:** 10.3389/fimmu.2025.1597282

**Published:** 2025-10-16

**Authors:** Roberto Depascale, Anna Ghirardello, Elisabetta Zanatta, Chiara Franco, Marisol Bracalenti, Federico Pettorossi, Mariele Gatto, Elena Treppo, Beatrice Moccaldi, Margherita Zen, Stefano Piaserico, Christian Ciolfi, Luca Quartuccio, Andrea Doria, Luca Iaccarino

**Affiliations:** ^1^ Rheumatology Unit, Department of Medicine DIMED, University of Padua, Padua, Italy; ^2^ Academic Rheumatology Centre, Department of Clinical and Biological Sciences, University of Turin, AO Mauriziano di Torino, Turin, Italy; ^3^ Rheumatology Unit, Department of Medicine DAME, University of Udine, Udine, Italy; ^4^ Dermatology Unit, Department of Medicine DIMED, University of Padua, Padua, Italy

**Keywords:** inflammatory myopathies, dermatomyositis, anti-SAE antibodies, refractory skin involvement, immunosuppresants

## Abstract

**Aim:**

We aimed to describe the clinical and serological characteristics of anti-small ubiquitin-like modifier-activating enzyme (SAE)-positive cases from a multicentric cohort of patients affected with idiopathic inflammatory myopathies (IIMs).

**Methods:**

Anti-SAE antibody-positive patients (determined by line immunoassay) from a prospective cohort of patients with IIM were retrospectively evaluated. We considered features at disease onset and during follow-up. Muscular involvement was evaluated by the Manual Muscle Test-8, creatine phosphokinase (CK) levels, and/or magnetic resonance imaging; interstitial lung disease (ILD) was evaluated by high-resolution computed tomography; and skin and joint involvement was evaluated by clinical judgment. The therapeutic approach was also reported in all patients, and a literature review was also provided.

**Results:**

Out of 170 patients with IIM, 10 (5.9%) were anti-SAE positive, all classified as having dermatomyositis; therefore, among 80 patients with dermatomyositis, the prevalence of anti-SAE antibodies was 12.5%. The female-to-male ratio was 9:1. The median time from onset of symptoms to diagnosis was 1 year (range 0–2 years), and the mean age at onset of symptoms was 55.5 years (range 34–77 years). All patients had skin manifestations, including photosensitive rash, heliotrope rash, and Gottron’s sign and/or papules (one with ulcerations). Refractory features requiring multiple lines of immunosuppressants were observed in 60% of cases. Four patients had arthritis and/or inflammatory arthralgia; four had muscular involvement, usually mild; and none had ILD. One patient had a history of malignancy. All patients were treated with glucocorticoids and received different immunosuppressants, including cyclophosphamide.

**Conclusions:**

All patients with anti-SAE antibody positivity were classified as having dermatomyositis, with severe and refractory skin manifestations in most cases. One case of malignancy was described; therefore, cancer screening should be warranted in all anti-SAE patients.

## Introduction

Dermatomyositis (DM) is a rare and multisystemic autoimmune disorder included in the large spectrum of idiopathic inflammatory myopathies (IIMs) (1), characterized by chronic inflammation in the skin and skeletal muscles (2). DM patients can be classified into different phenotypes, according to clinical features and myositis-specific antibody (MSA) positivity (1–4). Five mutually exclusive MSAs have been associated with DM: anti-Mi2, anti-melanoma differentiation-associated protein 5 (MDA5), anti-nuclear matrix protein-2 (NXP2), anti-transcriptional intermediary factor-1-γ (TIF-1-gamma), and anti-small ubiquitin-like modifier-activating enzyme (SAE). All of them have shown diagnostic and prognostic values (5–7). Anti-SAE antibodies were first reported by Betteridge et al. in 2007 (8) and then described in 2009 in a cohort of patients with IIM from the United Kingdom (9). Anti-SAE antibodies bind the small ubiquitin-like modifier (SUMO)-activating enzyme, characterized by two subunits (SAE1 and SAE2). SUMO is involved in post-translational modification of several target proteins by “sumoylation” (9). Sumoylation is an important regulator of the normal function of many proteins, which has been hypothesized to play an important role in the pathogenesis of some human diseases (10–12). The most common technique used for the detection of anti-SAE antibodies in patient sera is radiolabeled 35S protein immunoprecipitation (IP), but enzyme-linked immunosorbent assay (ELISA) and immunoblotting can also be applied (10). The prevalence of anti-SAE autoantibodies reported in the literature ranges from 1%–3% in Asians to 6%–8% in Caucasians (8, 10, 13, 14). The clinical phenotype of anti-SAE-positive DM patients is often characterized by amyopathic dermatomyositis at disease onset. Skin manifestations can be severe and often pruritic. Muscle disease, when present, is usually mild and often develops later during the disease course. Dysphagia appears to be another common manifestation among anti-SAE-positive patients. Interstitial lung disease (ILD), usually in the form of organizing pneumonia (OP), is another possible feature, often mild and subclinical. The prevalence of cancer varies among different studies (2, 15).

This study aims to describe the clinical features of patients with anti-SAE antibody positivity in a multicenter cohort of patients affected with IIM. A narrative review of cases reported in the literature will also be provided.

## Methods

Patients with anti-SAE positivity were retrospectively evaluated from a prospective multicentric cohort (Padua University Hospital and Udine University Hospital) of patients with IIM according to the EULAR/ACR and/or Bohan and Peter and/or European Neuromuscular Center (ENMC) classification criteria (16–18) since January 2011 to January 2025.

**Figure 1:**
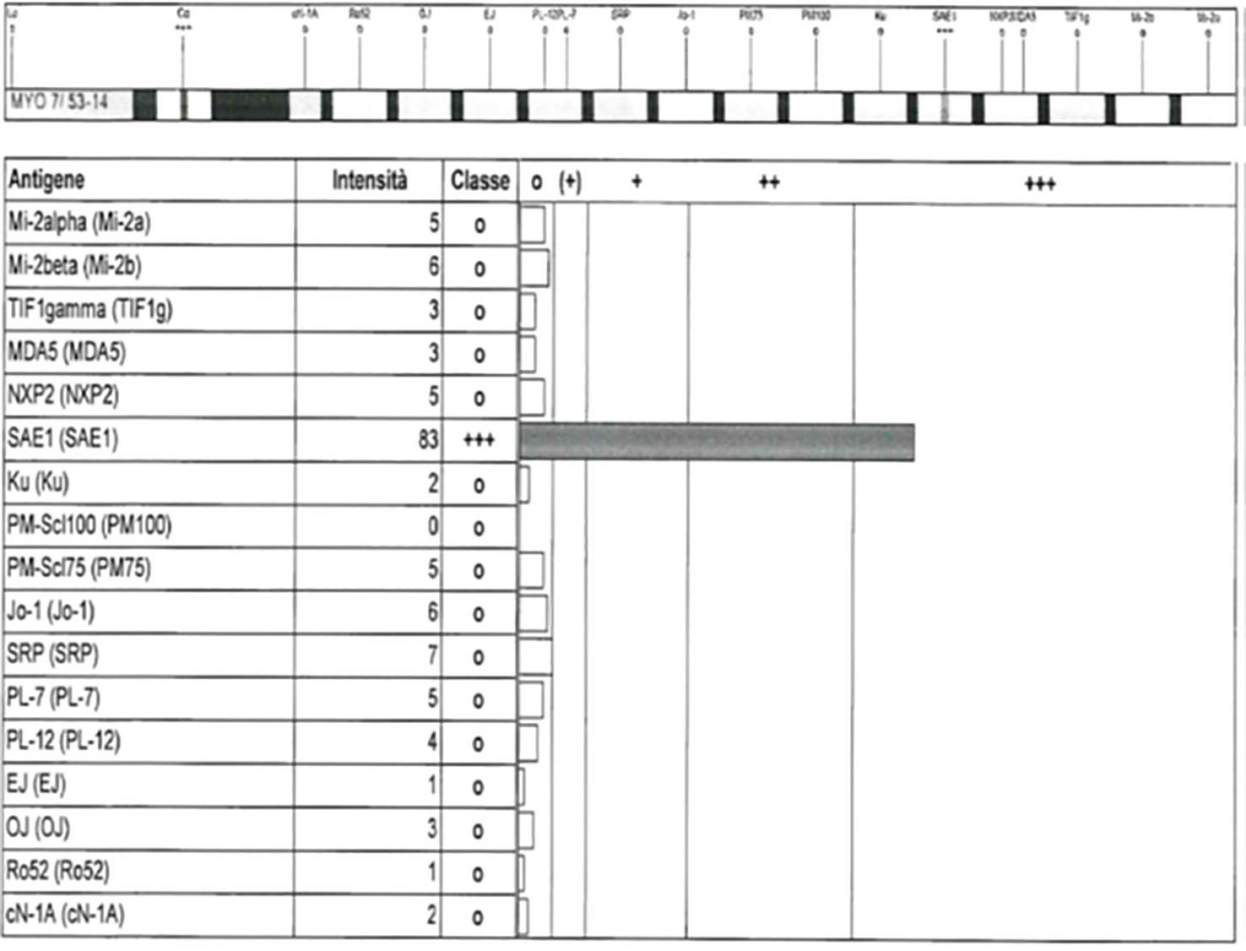
Representative image of anti-SAE-1 positivity in case 3 by a commercial line immunoassay (EUROLINE myositis profile 3).

Antinuclear antibodies (ANA) were detected through screening by indirect immunofluorescence (IIF), and specific MSA/MAA (myositis-associated antibodies) were tested using multiparametric line immunoassay according to the manufacturer’s protocol (EUROLINE, Lubeck, Germany). A representative image of anti-SAE-1 positivity in case 3 by a commercial line immunoassay is visualized in **Figure 1**.

In the prospective cohort, physical examination findings were obtained since the first visit. Among the laboratory tests, the closest values to the date of the visit were recorded. Muscle involvement was defined by muscular weakness assessed through the Manual Muscle Test-8 (MMT-8) and elevated creatine phosphokinase (CK) level (above the upper limit of normal) and/or muscular edema in T2-weighted images on muscle magnetic resonance imaging (MRI). All patients affected with DM underwent high-resolution computed tomography (HRCT) at baseline to assess the presence of ILD. Lung involvement was also evaluated during the follow-up according to the onset of new respiratory symptoms and/or restrictive pattern shown by pulmonary function tests (PFTs). Furthermore, all patients affected with DM were initially screened for cancer by using full-body computed tomography, esophagogastroduodenoscopy, and colonoscopy. Medications used by patients were also recorded, and response/refractoriness to treatment was defined by the physician’s judgment. Precisely, one patient was deemed refractory in case of inadequate response to glucocorticoids and at least two immunosuppressants.

The literature review was performed searching in PubMed, LiSSa, BDSP, and Cochrane Library databases for articles related to the association of DM and the anti-SAE autoantibody up until January 2025. We included all papers with anti-SAE DM case(s) description. We used the following keywords: anti-SAE, dermatomyositis, and skin in myositis.

The study was carried out in accordance with the Declaration of Helsinki and approved by our institution’s ethics committee (Azienda Ospedaliera di Padova, n. 5505/A/22).

## Results

A total of 170 patients with IIM were enrolled in the study. Among them, 10 (5.9%) were anti-SAE positive, all diagnosed with DM. The prevalence of anti-SAE antibodies in patients with DM of our cohort (*n*=80) was 12.5%. All patients were Caucasian and 90% were women. The mean age at disease onset was 55.5 years (range 34–77 years). The median time from onset of symptoms to diagnosis was 1 year (range 0–2 years), and the median follow-up duration was 35 months (range 23–58 months).

### Clinical features

The clinical and serological features of anti-SAE-positive patients are described in **Table 1**.

**Table 1:** Clinical and serological features of anti-SAE-positive patients at baseline and during follow-up.

Parameters		Case 1	Case 2	Case 3	Case 4	Case 5	Case 6	Case 7	Case 8	Case 9	Case 10
Age of onset (years)		34	61	45	32	45	57	77	45	64	50
Age at diagnosis (years)		35	61	47	32	45	58	78	45	64	50
Gender		F	M	F	F	F	F	F	F	F	F
Presentation	Fever	−	−	+	+	+	−	−	−	−	−
	Muscle weakness	−	+	−	−	+	−	+	−	−	−
	Myalgia	+	+	−	−	+	−	+	−	−	−
	Arthritis/arthralgias	−	−	+	+	+	+	−	−	−	−
	Voice change	−	−	−	−	+	−	−	−	−	−
	Dyspnea	−	−	−	−	+	−	−	−	−	−
	Dysphagia	−	−	−	+	+	−	+	−	−	−
	Diffuse rash	+	+	+	+	+	−	+	+	+	+
	Pruritus	+	+	−	+	+	+	−	+	+	+
	Heliotrope rash	+	+	+	+	+	+	+	+	+	+
	Gottron’s papules	+	+	+	+	+	+	+	+	+	+
	Calcinosis	−	−	+	−	−	−	−	−	−	−
	Skin ulcers	−	−	+	−	−	−	−	−	−	−
	Malignancy	−	−	−	−	−	+	−	−	−	−
	Interstitial lung disease	−	−	−	−	−	−	−	−	−	−
	Muscular edema (MRI)	+	−	NA	+	−	NA	NA	NA	NA	NA
	MMT-8 (baseline)	135/150	148/150	150/150	145/150	120/150	150/150	136/150	135/150	150/150	150/150
	MMT-8 (6 months)	140/150	145/150	NA	140/150	130/150	NA	135/150	NA	NA	NA
	MMT-8 (12 months)	140/150	142/150	NA	NA	NA	146/150	130/150	NA	NA	NA
Lab investigations	CK (U/L) (baseline)	NA	100	110	100	13,000	77	512	438	300	250
	CK (U/L) (6 months)	200	300	150	120	450	90	400	NA	NA	NA
	CK (U/L) (12 months)	NA	350	180	NA	NA	80	NA	NA	NA	NA
	AST (U/L) (baseline)	NA	27	49	52	300	27	NA	NA	NA	NA
	LDH (U/L) (baseline)	NA	70	80	386	NA	NA	339	NA	NA	NA
	ANA MSA/MAA	+ NA SAE-1	+ + SAE-1 PM-Scl-100	+ − SAE-1	+ − SAE-1 Tif-1-gamma PL-7 (borderline)	+ NA SAE	+ SSA SAE-1	+ SSA SAE-1	+ SAE-1	+ SAE-1	+ SAE-1
Refractoriness		+	−	+	+	+	+	+	−	−	NA

CK, creatine phosphokinase; AST, aspartate transaminase; LDH, lactate dehydrogenase; ANA, anti-nuclear antibody; MAA, myositis-associated antibodies; MSA, myositis-specific antibodies; MTX, methotrexate; MMT-8, Manual Muscle Test 8; NA, not available; +, present; −, absent.

**Figure 2:**
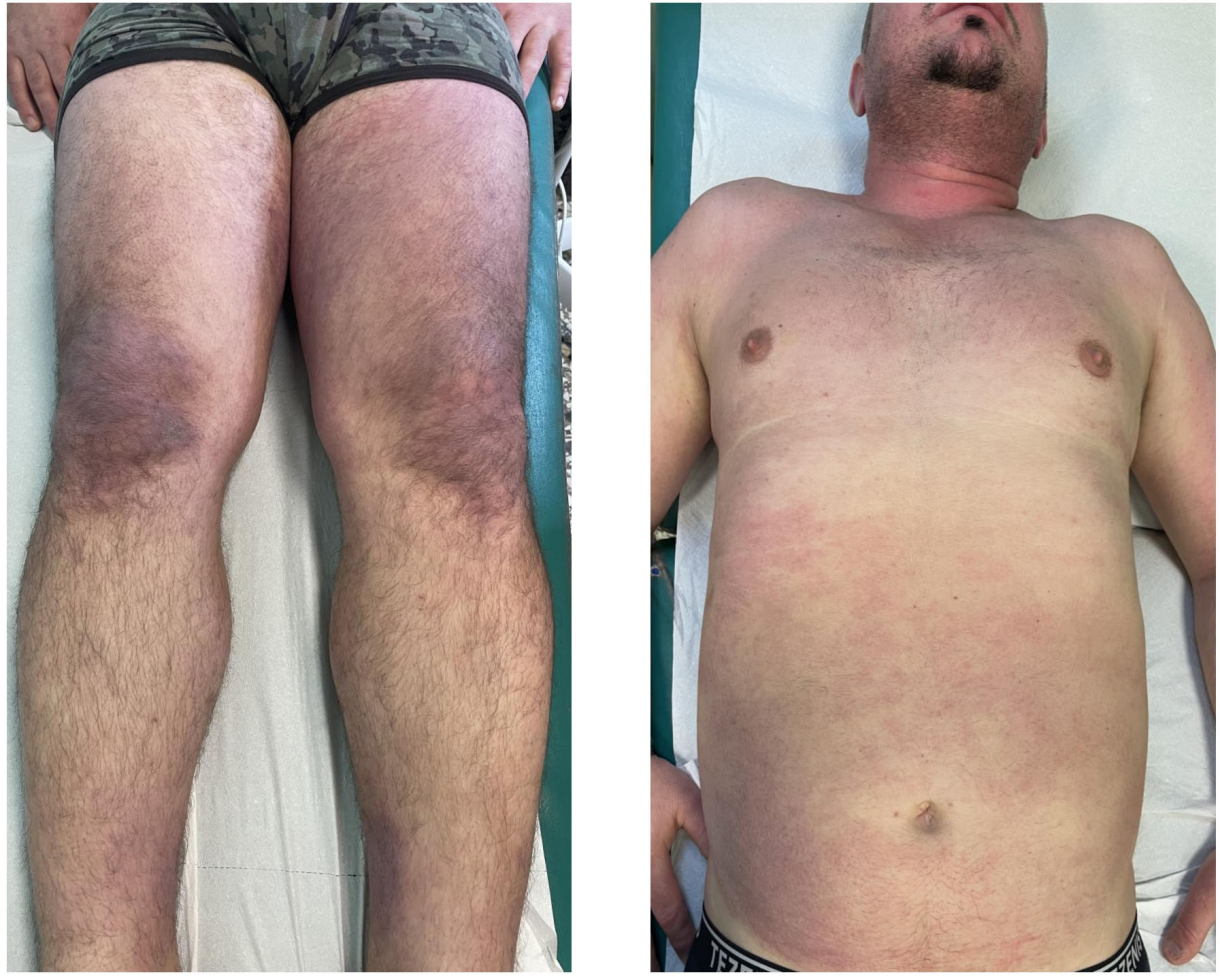
Case 2: A 62-year-old male patient diagnosed with anti-SAE+ DM. Wide violaceous erythematous plaques involving the knees, trunk, and hands. Consent obtained.

All patients displayed skin manifestations, most of them initially presenting with a diffuse rash (**Figure 2**). Gottron papules and Gottron’s sign were found in 100% of patients. One patient had a diffuse skin involvement associated with panniculitis and skin ulcerations. Seven patients underwent skin biopsy showing non-specific dermatitis. Calcinosis was described in one case involving the buttocks and thighs. None had mechanic’s hands. Muscle weakness occurred in three patients (30%). In most cases, the onset of myositis occurred after skin involvement, with a mean of 8 months (range 3–8 months). In two cases, not complaining of muscular weakness but only myalgia, edema on muscular MRI was found. Three patients had elevated CK. In one case, the elevation of muscular enzymes was mild, occurring 2years after the skin manifestations. The other patient developed an acute rhabdomyolysis requiring hospitalization with very high levels of CK and acute kidney damage at disease onset. All potential causes of rhabdomyolysis, including infectious and toxic etiologies, were ruled out, and the detection of autoantibodies supported an autoimmune origin.

Muscular biopsy was performed in one patient, confirming the histological pattern of DM. Three patients reported dysphagia. Arthritis or inflammatory arthralgias were reported in four cases (40%). No patient had clinical, functional, or radiological signs of ILD at baseline, and further signs or symptoms of pulmonary involvement were found during follow-up. As reported in **Table 1**, in addition to anti-SAE positivity, case 4 was positive for anti-TIF1 gamma and borderline positive for anti-PL-7. In such patients, borderline anti-tRNA synthetase positivity was apparently not related to lung involvement, both at diagnosis and follow-up.

There was one case of malignancy (10%) in our cohort. The patient was a 57-year-old lady diagnosed with DM, and cancer screening found a localized and differentiated colon adenocarcinoma. She underwent colon resection in 2020, remaining cancer-free thereafter.

### Treatment

Methotrexate (MTX) was the most commonly used medication in our cohort, together with glucocorticoids (70% and 100%, respectively). Six patients (60%) had a refractory cutaneous disease and required multiple medication changes, including one case with ulcers and panniculitis requiring cyclophosphamide (CYC), after failure of several immunosuppressants. Because of persistent cutaneous manifestations despite steroids and MTX, one patient was successfully treated with the JAK inhibitor baricitinib (BARI) (**Table 2**).

**Table 2:** Treatment timeline and prespecified assessments.

Case	Time-to-treatment (months from onset)	Initial glucocorticoid dose (mg/day)	Immunosuppressive treatment (lines)	Skin assessments (time points/months)	Muscle outcome assessments (time points/months)
1	12	25	1st MTX 7.5 mg 2nd MMF 2 g 3rd CsA 100 mg	0, 6, 12	0, 6, 12
2	6	25	1st MMF 2 g	0, 6, 12	0, 6, 12
3	24	50	1st MTX 10 mg 2nd MMF 2 g 3rd IVIg 0.4 g/kg/day 4th CYC 1 g	0, 6, 12	0, 6, 12
4	4	25	1st MTX 10 mg 2nd BARI 4 mg	0, 6, 12	0, 6, 12
5	6	50	1st MTX 15 mg 2nd IVIg 0.4/kg/day 3rd CsA 150 mg	0, 6, 12	0, 6, 12
6	12	37.5	1st MTX 7.5 mg 2nd IVIg 0.4/kg/day 3rd CsA 100 mg 4th RTX 1g ×2	0, 6, 12	0, 6, 12
7	12	12.5	1st MTX 7.5 mg + HCQ 200 mg 2nd IVIg 0.4/kg/day	0, 6, 12	0, 6, 12
8	3	37.5	1st HCQ 200 mg + MMF 2 g	0, 6, 12	0, 6, 12
9	6	25	1st MTX 10 mg	0, 6, 12	0, 6, 12
10	6	25	NA	0,6	0,6

Treatment timeline including time-to-treatment, initial glucocorticoid dose, immunosuppressive regimen (line, drug, dose, duration), and clinical subjective assessments for skin and muscle (MMT-8).

MTX, methotrexate; IVIg, intravenous immunoglobulin; RTX, rituximab; CYC, cyclophosphamide; CsA, cyclosporin A; HCQ, hydroxychloroquine; BARI, baricitinib; MMF, mycophenolate mofetil.

## Narrative literature review and discussion

To date, we have been able to find 208 anti-SAE adult patients reported in the literature (**Table 3**).

**Table 3:** Anti-SAE-positive DM patients reported in the literature.

Author, year country (reference)	Cases	Clinical manifestations	CAM
Betteridge et al., 2007 UK (8)	2	DM 100% (*n* = 2) Muscle weakness 100% (*n*=2) ILD 100% (*n* = 2) Dyspaghia 100% (*n* = 2)	None
Betteridge et al., 2009 UK (9)	11	DM 82% (*n* = 9) Muscle weakness 78% (*n* = 7) Dysphagia 78% (*n* = 7) ILD 18% (*n* = 2) Arthritis 18% (*n* = 2)	18% (*n* = 2) Cancer site not reported
Tarricone et al., 2012 Italy (10)	5	DM 100% (*n* = 5) Muscle weakness 100% (*n* = 5)	20% (*n* = 1) Ovarian cancer
Muro et al., 2013 Japan (22)	2	DM 100% (*n* = 2) Muscle weakness 100% (*n* = 2) ILD 50% (*n* = 1)	50% (*n* = 1) Colorectal cancer
Fujimoto et al., 2013 Japan (14)	7	DM 100% (*n* = 7) Muscle weakness 86% (*n* = 6) ILD 71% (*n* = 5) Dysphagia 29% (*n* = 2)	14% (*n* = 1) Colorectal cancer
Chen et al., 2015 China/Japan (26)	2	DM 100% (*n* = 2)	Not reported
Ge et al., 2017 China (19)	12	DM 100% (*n* = 12) Muscle weakness 67% (*n* = 8) ILD 64% (*n* = 7) Dysphagia 64% (*n* = 7)	18% (*n* = 2) Lung cancer
Lee et al., 2017 Australia (30)	1	DM 100% (1) Muscle weakness 100% (1) Skin ulcers 100% (1)	None
Inoue et al., 2018 Japan (36)	6	DM 100% (*n* = 7) Muscle weakness 85% (*n* = 6) ILD 42% (*n* = 3)	20% (*n* = 2) Colorectal, renal cancer
Peterson et al., 2018 USA (38)	19	DM 100% (*n* = 19) Muscle weakness 60% (*n* = 11)	6% (*n* = 1) Renal cancer
Zamora et al., 2019 Spain (31)	1	Muscle weakness 100% (*n* = 1) ILD 100% (*n* = 1) Myocarditis 100% (*n* = 1)	None
Matsuo et al., 2019 Japan (29)	1	DM 100% (*n* = 1) Muscle weakness 100% (*n* = 1) ILD 100% (*n* = 1) Dysphagia 100% (*n* = 1)	100% (*n* = 1) Colorectal cancer
Jia et al., 2019 China (21)	1	DM 100% (*n* = 1)	None
Gono et al., 2019 Japan (23)	2	DM 100% (*n* = 2) ILD 50% (*n* = 2) Dysphagia 50% (*n* = 2)	None
Zampeli et al., 2019 Greece (27)	6	DM 83% (*n* = 5) Dysphagia 50% (*n* = 3)	None
Betteridge et al., 2019 UK, Sweden, Hungary, Czech Republic (13)	42	DM 100% (*n* = 42) (further clinical features are not reported)	Not reported
Albayda et al., 2021 North America (25)	19	DM 95% (*n* = 18) Muscle weakness 53% (*n* = 10) Dysphagia 42% (*n* = 8) Arthritis 42% (*n* = 8) ILD 37% (*n* = 7)	26% (*n* = 5) Colorectal, renal, breast, lymphoma
Demortier et al., 2023 France (20)	49	DM 96% (*n* = 47) Muscle weakness 84% (*n* = 43) Dysphagia 39% (*n* = 19) ILD 21% (*n* = 10) Calcinosis 10% (*n* = 5)	16% (*n* = 8) Colorectal, melanoma, lung, ovarian, hematologic
Present study, 2023 Italy	10	DM 100% (*n* = 10) Muscle weakness 40% (*n* = 4) Arthritis 40% (*n* = 4) Panniculitis 10% (*n* = 1) Calcinosis 10% (*n* = 1) Rhabdomyolysis 10% (*n* = 1)	10% (*n* = 1) Colorectal
Fornaro et al., 2024 (46)	10	DM 70% (*n* = 7) Muscle weakness 80% (*n* = 8) Arthritis 20% (*n* = 2) Calcinosis 10% (*n* = 1) ILD 30% (*n* = 3)	20% (*n* = 2)

According to these studies, ethnic background may influence the frequency of disease manifestations (24, 25); Middle Eastern anti-SAE patients have a higher risk of developing cancer, ILD, dysphagia, and diffuse and pruritic erythema than Caucasian patients (26). Although disease symptoms may vary among ethnicities, the prevalence of skin, muscular, and lung manifestations is similar (27, 28). In our study, anti-SAE positivity was characterized by predominant diffuse and often pruritic skin manifestations, accompanied by clinical or subclinical myopathy that typically developed after the onset of skin lesions. These findings are in line with previous studies (10, 21, 25, 29). Interestingly, we also reported one case of severe cutaneous and subcutaneous involvement with panniculitis and necrotic ulcers requiring deep immunosuppressant treatment, as rarely described in the literature (30). Although patients with anti-SAE antibodies are usually classified as having an amyopathic form of DM, in our cohort, overt muscle disease was found in three patients (30%). This finding suggests that muscle involvement should be screened in all cases, particularly during the follow-up (25). Interestingly, we also described a case of acute and potentially fatal rhabdomyolysis at disease onset. Only another single case report of a patient with severe muscle and cardiac involvement (myocarditis), leading to death, was described (31). In our cohort, no sign of ILD was found (0%). Among anti-SAE-positive patients, evidence of preserved pulmonary functions and a higher prevalence of organizing pneumonia pattern rather than other MSAs has been reported in the literature (10, 14, 32).

The coexistence of more than one MSA, as found in case 4, can be observed by multi-analytic line immunoassays, as recently reported (11). In dermatomyositis, the presence of multiple autoantibody positivities frequently does not correspond to specific clinical manifestations. It may result from analytical artifacts or antigen cross-reactivity and lack a clear consensus for interpretation in clinically discordant cases. It highlights the need for further research to elucidate this phenomenon (11).

Finally, during cancer screening, one female patient from our cohort was diagnosed with non-metastatic colorectal adenocarcinoma. In the literature, 27 out of 208 patients (12.9%) with cancer-associated myositis in anti-SAE patients have been previously described (13, 19, 24, 25); however, the prevalence is underestimated because some papers did not evaluate or did not report any data regarding cancer association. In our cohort, all patients with DM, including anti-SAE-positive patients, underwent screening for neoplasms. In line with the reports in the literature, we therefore recommend screening for cancer in all patients with anti-SAE positivity (22, 33, 34).

Most patients affected with IIM respond well to glucocorticoids, although randomized clinical trials are still lacking. Nevertheless, a significant proportion of patients affected with IIM fail to respond to conventional immunosuppressants. Despite the overall good prognosis, difficult-to-treat skin disease might be an issue in the management of anti-SAE patients (35–38). As a matter of fact, in our cohort, 6 out of 10 patients (60% refractory rate) with refractory skin disease were given different immunosuppressants to control cutaneous disease activity, including CYC in a patient with severe cutaneous involvement and skin ulcers. Also, previous studies have pinpointed this feature of anti-SAE patients, reporting a percentage of difficult-to-treat and resistant skin manifestations in approximately 40% of patients (20, 25). Interestingly, a good response to BARI in the cutaneous domain was described in a young patient of our cohort. Among all IIM subtypes, growing evidence supports the role of interferon (IFN) in sustaining the pathogenesis of several manifestations in DM, particularly cutaneous disease (39). IFN signaling relies upon the Janus kinase-signal transducer and activator of transcription (JAK/STAT) cascade, which has become the target of the novel family of small-molecule JAK inhibitors in various diseases (40). Nowadays, the role of BARI in the management of IIM is under evaluation in two clinical trials (41, 42). Finally, despite the risk of infection, which should always be considered (43, 44), another option for refractory cases is the use of rituximab (RTX) (45), similar to one case from our cohort.

Our study has strengths and limitations. The main strength is that our patients were followed up prospectively with a long period of observation (mean 3 years); in addition, clinical and serological data were prospectively recorded at all visits; therefore, most of the patients had complete data for the study.

Limitations include the small number of patients described and the retrospective nature of the study. Furthermore, ethnicity can be a limitation, since all of our patients were Caucasian, and it may not reflect the heterogeneous characteristics of anti-SAE patients among different countries (20). The absence of ILD cases among anti-SAE patients in our series should be interpreted with caution. Although anti-SAE dermatomyositis typically shows lower ILD prevalence than other subsets, such as anti-MDA5, larger cohorts and longer follow-up are required to precisely estimate ILD risk (9).

Another limitation is that, in our cohort, only one patient underwent muscular biopsy for the histological confirmation of inflammatory myositis. On the other hand, current EULAR guidelines (16) recommend muscle biopsy in patients with presumed IIM, but it is not mandatory when cutaneous manifestations and serological characteristics are strongly suggestive of DM (3).

## Conclusions

Amyopathic or hypomyopathic DM is the most common clinical presentation of patients with anti-SAE positivity enrolled in our cohort. Skin involvement is severe and refractory in most cases and requires multiple lines of immunosuppressive therapy. Muscular involvement is usually mild but tends to develop during follow-up. Finally, given the limited number of cases and the current gaps in knowledge, future research should aim to identify reliable biomarkers that can better define the clinical spectrum, predict disease course, and guide therapeutic decisions in anti-SAE dermatomyositis.

## Data Availability

The original contributions presented in the study are included in the article/supplementary material. Further inquiries can be directed to the corresponding author.
